# Cavernome du quatrième ventricule

**DOI:** 10.11604/pamj.2017.26.101.11580

**Published:** 2017-02-26

**Authors:** Youssef Alaoui Lamrani, Mustapha Maâroufi

**Affiliations:** 1 Faculté de Médecine et de Pharmacie, Université Sidi Mohamed Ben Abdellah Fès, Service de Radiologie, CHU Hassan II, Fès, Maroc

**Keywords:** Hémangiome caverneux, quatrième ventricule, IRM

## Image en médecine

Monsieur KR, patient de 58 ans, admis pour trouble de l’équilibre avec diplopie horizontale gauche installés la veille de son admission. Devant cette atteinte vestibulaire et paralysie du VI gauche, une IRM a été effectuée attestant d’un cavernome intraventriculaire (CIV), rarement décrit au V4. Les cavernomes représentent des hamartomes pouvant atteindre tout le névraxe. IRM cérébrale montrant une lésion nodulaire (flèche) du plancher du 4^ème^ ventricule (V4), en hypersignal T1 (a), hyposignal annulaire en T2 (b) et T2* (c). La séquence injectée T1 montre une anomalie veineuse de développement (AVD) paraventriculaire gauche (étoile). La prévalence des CIV varie entre 2,5% et 10%, leurs localisations intraventriculaires intéressent le 3^ème^ ventricule, ventricules latéraux, trigone, et V4, dans 44%, 27%, 20% et 9% respectivement. Leurs manifestations cliniques sont aspécifiques liées essentiellement à l’hypertension intracrânienne. L’imagerie des CIV est similaire aux localisations intraparenchymateuses avec comme particularité, une tendance à des tailles plus volumineuses du fait d’une croissance non contrebalancée par le LCR. La TDM montre une lésion ronde hyperdense, avec calcifications annulaires en “popcorn” assez caractéristiques. Quant à l’IRM, le CIV est hétérogène en T1, en hypersignal central en T2 avec un anneau périphérique d’hyposignal relatif au dépôt d’hémosidérine. La séquence T2* est intéressante dans la détection de cavernomes multiples. Les séquences injectées peuvent montrer des AVD fréquemment associées aux cavernomes. Les diagnostics différentiels (DD) des CIV incluent astrocytomes de bas grade, neurocytomes, et tératomes. L’hypersignal central et l’anneau périphérique d’hyposignal T2, rendent le diagnostic de cavernome assez aisé et éliminent les DD.

**Figure 1:**
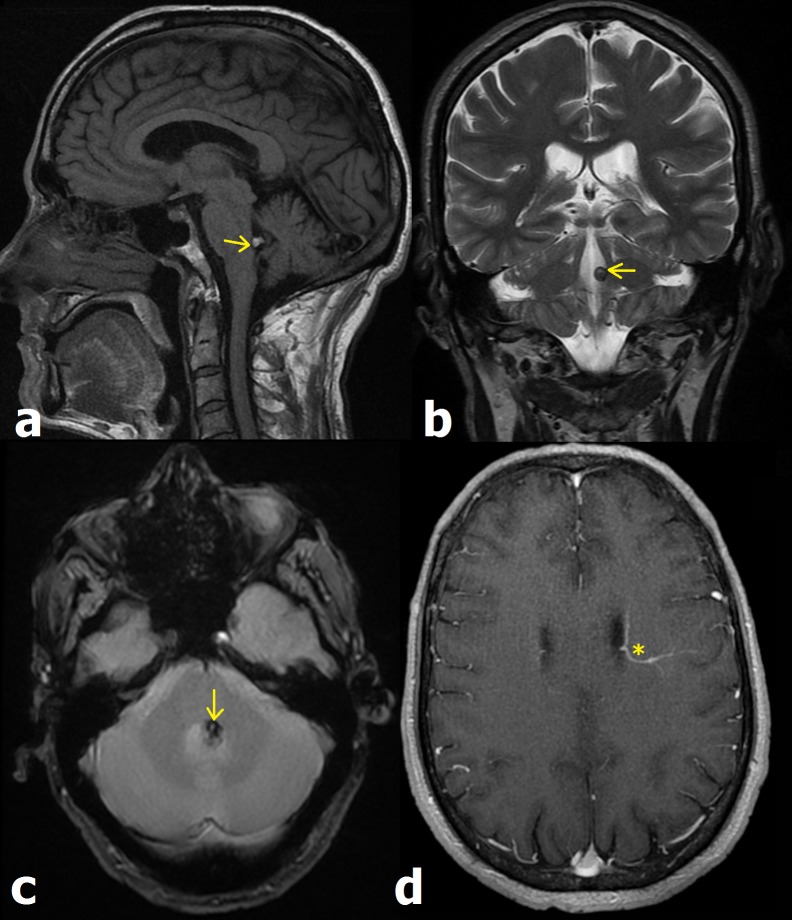
IRM cérébrale montrant une lésion nodulaire (flèche) du plancher du 4ème ventricule (V4), en hypersignal T1 (a), hyposignal annulaire en T2 (b) et T2* (c). La séquence injectée T1 montre une anomalie veineuse de développement (AVD) paraventriculaire gauche (étoile)

